# Estimation of Distributed Fermat-Point Location for Wireless Sensor Networking

**DOI:** 10.3390/s110404358

**Published:** 2011-04-13

**Authors:** Po-Hsian Huang, Jiann-Liang Chen, Yanuarius Teofilus Larosa, Tsui-Lien Chiang

**Affiliations:** 1 Department of Computer Science and Information Engineering, Yuanpei University, No. 306, Yuanpei Street, Hsinchu, Taiwan; 2 Department of Electrical Engineering, National Taiwan University of Science and Technology, Taipei, Taiwan; E-Mails: Lchen@mail.ntust.edu.tw (J.-L.C.); larosa@ee.ntust.edu.tw (Y.T.L.); 3 Department of Computer Science and Information Engineering, National Dong Hwa University, Hualien, Taiwan

**Keywords:** wireless sensor network, distributed fermat-point location estimation (DF-PLE), bounding box algorithm

## Abstract

This work presents a localization scheme for use in wireless sensor networks (WSNs) that is based on a proposed connectivity-based RF localization strategy called the distributed Fermat-point location estimation algorithm (DFPLE). DFPLE applies triangle area of location estimation formed by intersections of three neighboring beacon nodes. The Fermat point is determined as the shortest path from three vertices of the triangle. The area of estimated location then refined using Fermat point to achieve minimum error in estimating sensor nodes location. DFPLE solves problems of large errors and poor performance encountered by localization schemes that are based on a bounding box algorithm. Performance analysis of a 200-node development environment reveals that, when the number of sensor nodes is below 150, the mean error decreases rapidly as the node density increases, and when the number of sensor nodes exceeds 170, the mean error remains below 1% as the node density increases. Second, when the number of beacon nodes is less than 60, normal nodes lack sufficient beacon nodes to enable their locations to be estimated. However, the mean error changes slightly as the number of beacon nodes increases above 60. Simulation results revealed that the proposed algorithm for estimating sensor positions is more accurate than existing algorithms, and improves upon conventional bounding box strategies.

## 1. Introduction

A wireless sensor network is a large-scale *ad hoc* wireless network of hundreds or even thousands of sensor nodes [1,2]. These sensor nodes are subject to power and computation capacity constraints and have many functions for monitoring various environmental conditions and for collecting highly-precise data, such as light, humidity, temperature, magnetism, acoustics, pressure and voice-level information [3,4]. Ongoing challenges in wireless sensor networking include the problem of obtaining location information by sensor nodes that are not equipped with specialized hardware (GPS, ultra-sound, acoustic and laser radiation). In fact, applications such as environmental monitoring and targeting tracking require sensor location information, and several fundamental techniques developed for wireless sensor networks also require sensor node location information. Therefore, location awareness is essential in wireless sensor networks.

Numerous sensor network applications require location awareness, whereas in sensor networks, nodes are deployed into an unplanned infrastructure in which no *a priori* knowledge of location exists [5,6]. Thus, a node must know its location in sensor networks. Generally, using a GPS position is an immediate solution. However, it is typically too expensive to incorporate a GPS receiver into a sensor node. Hence, localization schemes for sensor networks typically use a small number of seed nodes (beacons or anchors) that know their location and protocols whereby other nodes estimate their location based on the messages they receive.

Several localization strategies have been proposed, ranging from solutions dependent on hardware support by GPS and the presence of an established infrastructure, to range-free solutions that utilize signal strength, hop count to known landmarks or a priori knowledge about density of nodes in a network. Most of these strategies share a common feature: they use beacon nodes that know their own locations. Other sensor nodes identify their locations based on information provided by these beacon nodes. Furthermore, in localization techniques, centralized localization approaches depend on sensor nodes transmitting data to a central location, where computation is performed to determine the location of each node. Consequently they generate high communication costs and inherent delay. However, distributed localization schemes do not require centralized computation and each node determines its location using limited communication with nearby nodes. For instance, beacon-based distributed algorithms such as diffusion, bounding box, gradient multi-literation and APIT, typically start with a group of beacons. Nodes in the network obtain a distance measurement to a few beacons, and then use these measurements to estimate their locations.

**Figure 1.**
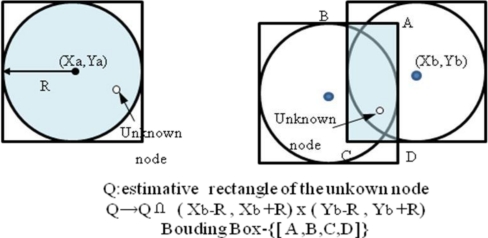
Bounding Box Algorithm.

One of the most well-known localization methods for WSNs is Convex Position Estimation (CPE) proposed by Dohetry *et al.* [7]. The CPE strategy is a computationally simple approach for localizing nodes when their ranges to several beacons are known. Notably, each node assumes that it lies at the intersection of the bounding boxes of its beacons. Further, the center of the bounding box is considered the approximate initial position of a sensor node. The accuracy of the bounding box approach is best when the actual positions of nodes are closest to the centers of their beacons. The CPE strategy utilizes a mechanism for bounding the feasible set with a rectangle parallel to the axes, and the algorithm can be run numerous times. It eventually obtains the smallest rectangle that bounds the feasible set (Figure 1). Although a feasible set can solve linear programming and semi-definite programming, unfortunately the solution may not be *optimal*. Further, the computational price of finding four points that define the tight rectangular upper bound for a feasible point is high, especially in large wireless sensor networks. However, its critical weakness is that random guessing causes very large mean errors in the network.

To overcome the disadvantages of CPE, in this paper DFPLE is proposed to minimize the mean error and computational price in estimating WSNs location. Like CPE, DFPLE is based on a bounding box algorithm to estimate the candidate of location. DFPLE expands the bound for location estimation using three cases of beacon node positioning. Therefore unlike the CPE has four bound points, DFPLE has dynamic number of bound points. Then, instead of using linear programming to find smaller area of estimation, DFPLE exploits the capability of Fermat Point calculation to refine the area of feasible set of solution and finally achieves minimal computational price.

The rest of this paper is organized as follows. Section 2 discusses existing location discovery algorithms, including range-based and range-free schemes. Section 3 then describes the proposed DFPLE algorithm. Next, Section 4 summarizes the performance analysis and simulation. Conclusions are finally drawn in Section 5, along with recommendations for future research.

## 2. Related Works

Several effective location discovery protocols for wireless sensor networks have been proposed in recent years [8–14]. Most solutions for location discovery in sensor networks require some nodes, or “beacons” (also called anchors or reference points), that use GPS or a manual configuration to obtain location awareness. Based on the technology used for location discovery, localization schemes can be classified as range-based and range-free. The former depends on the range information (e.g., absolute point-to-point distance information or directional information) needed to obtain nodes locations whereas the latter does not require range information.

### 2.1. Range-Based Schemes

Range-based schemes use absolute point-to-point distance or angle information to calculate locations between neighboring sensors. Such schemes estimate the absolute distance between a sender and receiver according to received signal strength or by time-of-flight of a communication signal. Common approaches for distance/angle estimation include Time of Arrival (ToA), Time Difference of Arrival (TDoA), Angle of Arrival (AoA) and Received Signal Strength (RSS). The accuracy of such estimation methods, however, depends on the transmission medium and surrounding environment, and they usually require complex hardware.

The Receiver Signal Strength Indicator (RSSI), initially used for power control in wireless networks, can also serve as a tool for distance estimation. The idea is that given a predefined transmission power, a signal propagation model that maps transmission power and a distance to a received power, one can estimate the distance from a receiver to a sender by identifying the strength of received power. The benefit of using RSSI for localization in sensor networks is obvious: trilateration can be achieved for all nodes using only three beacons, and nodes only perform passive listening. Unfortunately, existing signal propagation models are lacking, thereby significantly limiting localization accuracy; a receiver usually needs to perform sophisticated algorithms to synthesize the RSSI values from multiple senders to achieve adequate accuracy.

Time of Arrival (ToA) and Time Difference of Arrival (TDoA) measure signal arrival time or the difference in arrival times to calculate distance based on transmission time and speed. They can be applied to many different kinds of signals such as RF, acoustic and ultrasound signals. The ToA is less accurate than TDoA as processing delays and non-LOS (Line-of-Sight) propagation can generate errors. The ToA also requires synchronization to accurately measure time-of-flight.

The TDoA utilizes signal propagation speed, which is more robust than the signal attenuation characteristic used by RSSI. Ideally, when a sender and receiver are synchronized, the Time-of-Flight (ToF) measurement is already sufficient to identify the distance between the receiver and sender. However, synchronization, whose precision can match the radio signal speed, is hard to achieve. The TDoA mechanism is frequently utilized in cellular networks for localizing a handset. Since it only requires that the difference between arrival times is observed at several receivers, TDoA eliminates the need for synchronization between a handset and receivers. However, the receivers must be synchronized. Recent literature has defined TDoA as the difference between arrival times of two signals. This definition for TDoA actually applies to ToF, as the propagation time of one signal is measured and another signal is utilized for time synchronization. Employing different signal types for ranging has the limitation that one of them may not work properly in an environment that favors another. Therefore, ranging mechanisms relying on only one signal could be desirable for sensor location surveys.

**Figure 2.**
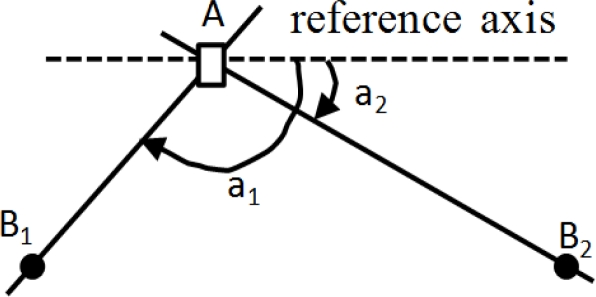
AoA Measurement.

The Angle of Arrival (AoA) scheme requires measurement of the angle at which a signal arrives at a base station or a sensor. It is used initially in cellular networks that require each receiver is equipped with additional gear (e.g., an antenna array) to detect the bearing of a sender’s signal. Figure 2 shows two position aware nodes, say *B_1_* and *B_2_* that are required to determine the position of a node *A*. Nodes *B_1_* and *B_2_* must be able to determine the direction from which a signal is coming. This can be achieved with an array antenna. An imaginary line is drawn from *B_1_* to *A* and another imaginary line is drawn from *B_2_* to *A*. The angle of arrival is defined as the angle that each of these lines makes with a line directed towards a common reference. The point at which these lines intersect determines the position of *A*. However, when *A*, *B_1_* and *B_2_* are all on the same straight line, another independent measurement is required to resolve the ambiguity. Accuracy of the AoA scheme is largely dependent on beam-width of antennas. Therefore, sensor nodes that must be localized are generally very small, and applying this mechanism is unrealistic due to the limitation on size and power consumption of a node.

### 2.2. Range-Free Schemes

In range-free localization schemes, the nodes determine their location without time, angle, or power measurements. Therefore, hardware design is dramatically simplified; however, such schemes are there- fore extremely cost effective. In such schemes, errors may be masked by network fault tolerance, redundancy computation, and aggregation. Bulusu proposed an outdoor localization scheme “Centroid”, an outdoor location scheme in which the nodes determine the location as the centroid of its proximate anchor nodes [15]. Compared to other schemes, the Centroid method is easier to implement and requires less overhead but is less accuracy. Niculescu and Nath proposed DV-hop, in which each node uses a distance vector-like approach to determine the number of hops to nodes with known locations, which are called “landmarks” [16]. Once the number of hops to at least three landmarks is known, the nodes determine the distance to landmarks by estimating average hop size and then determine absolute locations by applying multilateration. The Approximate Point in Triangle (APIT) mechanism resolves the localization problem by dividing the environment into triangular regions between anchor nodes [17]. By using a point-in-triangle test to determine its location relative to triangles formed by anchors, a node can reduce the size of its estimated location. The APIT mechanism defines the center of gravity of the estimated node location as the intersection of all triangles in which a node resides.

## 3. Distributed Fermat Point Location Estimation Algorithm

This section describes how the proposed DFPLE strategy uses the Fermat point of the triangle for an irregular wireless sensor network [18]. For simplicity, only a 2-D network is discussed. First, some assumptions are required for wireless sensor networks:

- There are *N* sensor nodes in the wireless sensor network.
- Every sensor node has a unique ID.
- Sensor nodes are deployed randomly.
- There are *M* beacon nodes in the network, where *0 < M < N*.
- Each beacon node is equipped with a GPS and, thus, knows its own location.
- The other (*N − M*) nodes are normal nodes that are unaware of their positions.
- For sake of effective-performance, the transmission power of a beacon node is modulated by the variable radius method. That is, the power level of beacon nodes can be modulated to high power level up to increase the communication range of beacon nodes to *2r,* where *r* is the transmission radius of normal nodes.

The DFPLE consists of four main phases on its operation: gathering beacon node location phase, estimating location, refining estimated location and error estimation. Each step of phases described as follows:

### **[Phase I]** Gathering Beacon Node Location

1. To gather information about other beacon nodes within communication range, beacon nodes must increase power to extend their communication range to *2r*.
2. The beacon nodes gather the ID and location information of neighboring beacon nodes by exchanging beacon frames.

### **[Phase II]** Location Estimation

**Figure 3.**
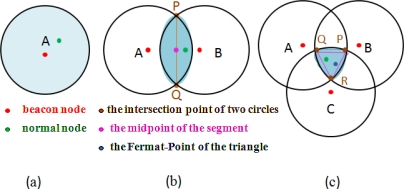
DFPLE Operation for Sensor Location Estimation.

1. Beacon nodes reduce power to their original level.
2. Normal nodes record all neighbors (including normal node ID, beacon node ID, and locations) within communication range.
3. Neighboring beacon nodes provide other beacon node locations, which are collected in Phase I. When the beacon node is beyond the communication range of normal nodes, the neighboring beacon node that is farthest from the normal beacon node is considered the beacon node.
4. The location of the normal node must meet one of the following three cases:
  - When the normal node is within communication range of a beacon node, the location of the beacon node is considered the most likely solution [see Figure 3(a)].
  - When the normal node is within communication range of two beacon nodes, the midpoint of the intersection of their communication ranges is considered the most likely solution [see Figure 3(b)].
  - When the normal node is within communication range of three beacon nodes, the Fermat point of the triangle which is formed by the intersection of the three circles in which the center of the circles are the beacon node locations is considered the most likely solution [see Figure 3(c)].

**Figure 4.**
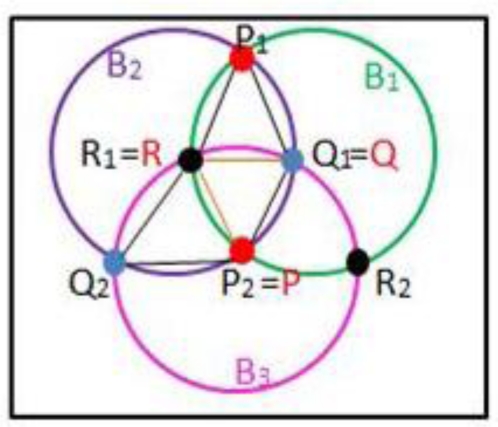
Calculate the Vertices in a Triangle.

Two intersecting circles must have two intersection points. Of course, three circles intersecting circles must have six intersection points. Therefore, a rule is needed for selecting the correct three intersection points needed to construct a triangle. The symbols are given by Table 1 for all intersection points. The rule works in three steps (see Figure 4):

Step 1: If $\bar{P_{1}Q_{1}}+\bar{P_{1}Q_{2}}>\bar{P_{2}Q_{1}}+\bar{P_{2}Q_{2}}$, then *P = P_2_* otherwise *P = P_1_*
Step 2: If $\bar{PQ_{1}}>\bar{PQ_{2}}$, then *Q = Q_2_* otherwise *Q = Q_1_*
Step 3: If $\bar{PR_{1}}>\bar{PR_{2}}$, then *R = R_2_* otherwise *R = R_1_*

**Figure 5.**
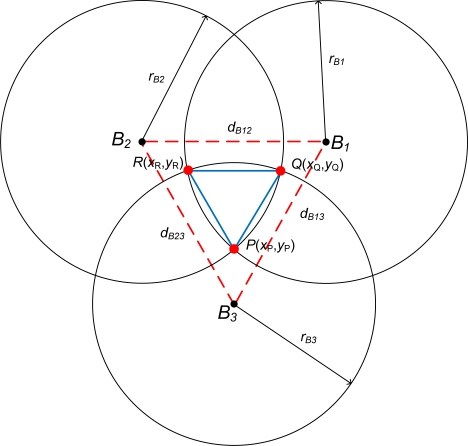
Estimation Locations of Three Beacons.

The calculations of vertices *P*, *Q*, and *R* coordinates are performed by simple geometric computation. Figure 5 depicts three neighboring beacon nodes (*B_n_*) circles with radius *r_n_* form three intersections. The distances from one beacon to the others are assumed differ (*d_B12_ ≠ d_B13_ ≠ d_B23_*). For two circles of *B_1_* and *B*_2_ intersect at *P*, the *x* and *y* coordinates *P* are approximately given by:

(1) $$x_{P}=\frac{r_{B1}^{2}-r_{B2}^{2}+d_{12}^{2}}{2d_{B12}},\;y_{P}=\frac{1}{2d_{B12}}\sqrt{2d_{12}^{2}\left(r_{B1}^{2}+r_{B2}^{2}\right)-\left(r_{B1}^{2}-r_{B2}^{2}\right)-\frac{d_{B12^{2}}}{4}}$$

From *step 1 of Phase I, r_B1_ = r_B2_ = r_B3_ = r*. Hence, the coordinates of vertices *P*, *Q*, *R* can be obtained using:

(2) $$\begin{array}{ll} x_{P}=\frac{d_{B12}}{2}, & y_{P}=\sqrt{r^{2}-\frac{d_{B12^{2}}}{4}}\\ x_{Q}=\frac{d_{B23}}{2}, & y_{Q}=\sqrt{r^{2}-\frac{d_{B23^{2}}}{4}}\\ x_{R}=\frac{d_{B13}}{2}, & y_{R}=\sqrt{r^{2}-\frac{d_{B13^{2}}}{4}} \end{array}$$

### [**Phase III]** Determine FERMAT Point

**Figure 6.**
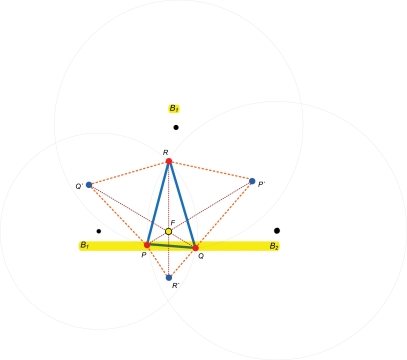
Formation of Three Virtual Triangles associated with Fermat Point.

The FERMAT point is point in Δ*PQR* that minimizes |*FP*| + |*FQ*| + |*FR*| (Figure 6). When all angles of Δ*PQR* are less than 120°, a unique Fermat point *F* lies inside the triangle such that $\bar{\mathrm{FP}}$, $\bar{\mathrm{FQ}}$ and $\bar{\mathrm{FR}}$ meet each other at mutual angles of 120°. The Fermat point is found as follows.

1. Construct a virtual equilateral triangle associated with each *PR*, *RQ*, and *QP*, designated Δ*PQ′R*, Δ*RP′Q*, and Δ*QR′P* respectively.
2. Construct lines *PP′*, *QQ′* and *RR′*. These are straight lines that connect the vertices of the triangle with the opposite vertices of the drawn virtual triangles.
3. Finally, *PP*`, *QQ′* and *RR′* intersect at the Fermat point, for which the sum of the distances from the point to the vertices of Δ*PQR* is minimal.

### [**Phase IV]** Refining Estimated Location

**Figure 7.**
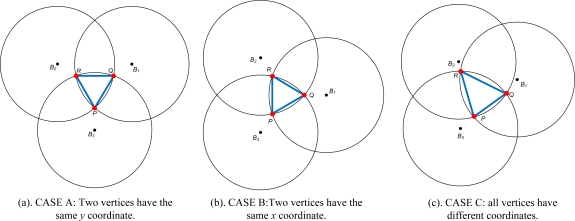
Cases of Δ*PQR* vertices position.

Δ*PQR* needs to be shrunk to reduce the error in the estimated location. When Δ*PQR* is constructed from three neighboring beacon nodes, two vertices may have the same *x* or *y* coordinate. Figure 7 presents three constructions of Δ*PQR*—cases A, B, and C. Each case is treated with respect to refinement of the estimated location.

**Figure 8.**
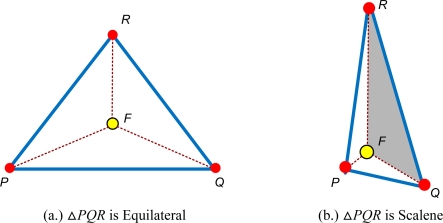
Types of Δ*PQR* Refinement.

The centroid of the triangle (*C*) can be considered to be a reference point to shrink the Δ*PQR*. However when centroid is used as refinement point, the area of Δ*PCQ,* Δ*PCR,* and Δ*PCQ* are always equal. Therefore using Fermat point provides advantages over centroid in providing a dynamic space to estimate the location of normal node. Since Fermat point yields different size of Δ*PFQ,* Δ*PFR,* and Δ*PFQ* the refinement of Δ*PQR* will accurately estimating the location of sensor nodes. In cases A and B, Δ*PQR* is an equilateral triangle, so the Fermat point is located at its center. The areas Δ*PFR =* Δ*PFQ =* Δ*QFR,* therefore Δ*PQR* need not be refined. In case C, Δ*PQR* is a scalene triangle so the areas Δ*PFR ≠* Δ*PFQ ≠* Δ*QFR*. Δ*PQR* area needs to be refined. Hence, the refinement of estimated location is performed by choosing the largest area of the triangles. Figure 8(b) shows the example where the largest triangle is Δ*QFR*. Therefore the estimated location is reduced to the Δ*QFR* area.

### **[Phase V]** Error Estimation

Localization accuracy is determined based on the closeness of a best estimate for the actual position of an unknown node. The closeness of a position estimate to the actual estimate is positively correlated with the accuracy of the algorithm. In this study, performance of the DFPLE algorithm is defined as the mean error (*μ*) from the computed to the actual unknown positions. *μ* given by Equation 3, provides a measure of the size of the feasible set:

(3) $$\mu =\frac{1}{n-m}\sum_{r=m+1}^{n}\sqrt{{\left(x_{\mathrm{est}}^{k}-x_{\mathrm{real}}^{k}\right)}^{2}+{\left(y_{\mathrm{est}}^{k}-y_{\mathrm{real}}^{k}\right)}^{2}}$$

*n* is the number of sensor nodes and m is the number of beacon nodes. $x_{\mathrm{real}}^{k}$ and $y_{\mathrm{real}}^{k}$ are the actual coordinates of the normal node with sensor ID *k*, while $x_{\mathrm{est}}^{k}$ and $y_{\mathrm{est}}^{k}$ are estimated coordinates of the normal node with sensor ID *k*. This phase utilized the characteristic of the Fermat point inside the triangle; namely, the Fermat point is the point at which the sum of its distances from vertices in a triangle is a minimum, to elevate the location estimation accuracy for the randomly chosen case in terms of mean error. Furthermore, the normal nodes estimate locations using simply arithmetical computation.

## 4. Performance Analysis

**Figure 9.**
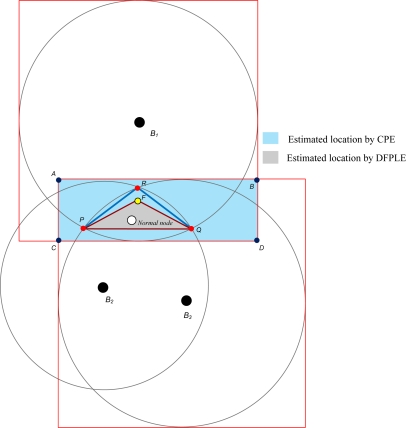
Location Estimation of DFPLE and CPE.

**Figure 10.**
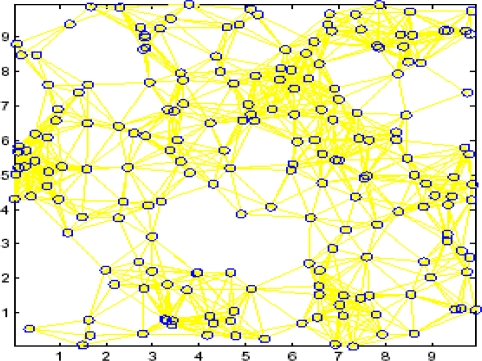
Simulation Environment.

The proposed DFPLE strategy was simulated using MATLAB in a static wireless sensor network. This simulation was conducted in a 2-D square area (5r5*r* and 10*r*10*r*) in which sensor nodes were randomly deployed. The DFPLE strategy was compared with the Convex Position Estimation (CPE) strategy to investigate whether the DFPLE algorithm achieves better accuracy and stability. Figure 10 shows a simulation environment in which 200 nodes were randomly distributed in a 10*r*10*r* square using a radio range of 1.5*r*. Compared to the CPE algorithm, the DFPLE algorithm achieves more accurate location estimation (Figure 9).

Most proposed location estimation algorithms generate position estimate errors. Even in idealized setups with no obstacles or external factors, relatively small errors from noisy sensor measurements can induce considerably larger errors in node position estimates. Such errors are related to a set of attributes that in this study are network setup attributes. Network setup attributes include the measurement technology used, accuracy of measurement technology used, network density, uncertainties in beacon node locations and beacon node densities. The simulations focus on the impact of three factors: density of sensor nodes, ratio of beacon nodes and response rate.

### 4.1. Sensor Node Density

**Figure 11.**
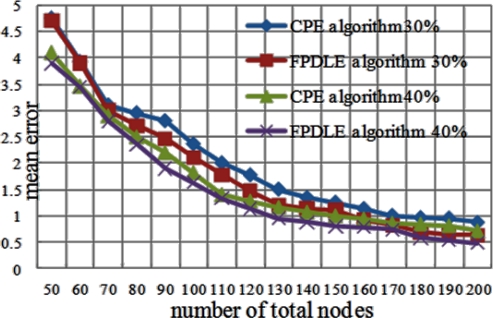
Node Density *vs.* Mean Error.

The impact on sensor node density is evaluated by increasing the number of sensor nodes from 50 to 200 in a fixed square area (10*r*10*r*). This experiment is conducted with 30% beacon nodes and 40% beacon nodes. Figure 11 shows the impact of node density on mean error. When the number of total nodes is below 150, the mean error decreases rapidly as node density increases. When the number of total nodes exceeds 170, mean error remains below 1%, and the impact of node density on mean error is minimal.

### 4.2. Sensor Node Density

**Figure 12.**
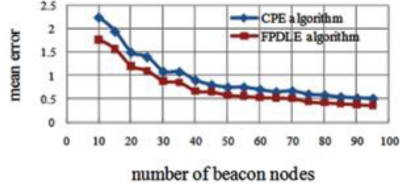
Number of Beacon Nodes *vs.* Mean Error.

The impact on the ratio of beacon nodes is evaluated by increasing the number of beacon nodes from 10 to 95 in a 10*r*10*r* square random deployment of 200 sensor nodes. Figure 12 shows the impact of the ratio of beacon nodes on mean error for the two algorithms.

### 4.3. Response Rate

**Figure 13.**
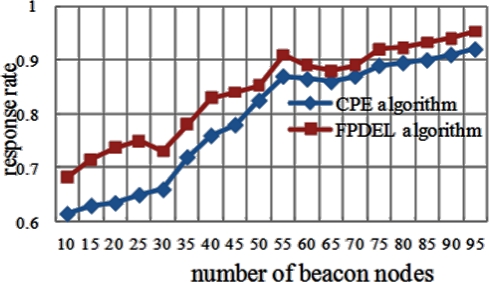
Number of Beacon Nodes *vs.* Mean Error.

In wireless sensor networks, response rate is also a metric for network performance. When a beacon node sends a query packet to its neighbors inside its communication range, if its neighbor’s location estimation is accurate, this neighbor must respond with a message sent back to the beacon node as soon as it receives the query package. Finally the response rate is calculated. The DFPLE algorithm has an acceptable performance when the number of beacon nodes exceeds 55 (Figure 13).

As mentioned in simulation results, the proposed DFPLE algorithm improves mean error for the randomly selected case of the existing bounding box algorithm (CPE strategy). Specifically, the proposed DFPLE algorithm has better performance than CPE algorithm in terms of location estimation.

## 5. Conclusions

This paper has presented DFPLE (Distributed Fermat-point Location Estimation) for WSNs. The proposed method of estimating sensor positions applies a Fermat point algorithm to estimate sensor node positions. Unlike the traditional bounding box algorithm, DFPLE is based on the shortest path from intersection between beacon nodes coverage area. The intersection vertices form a triangle which Fermat point is located to refine the estimated location of sensor nodes. The simulation, comparing DFPLE and CPE, revealed the effects of varying the number of sensor nodes and the proportions of beacon nodes. Simulation results demonstrate that the DFPLE algorithm for estimating sensor positions is more accurate than existing algorithms and improves upon conventional bounding box strategies.

## Figures and Tables

**Table 1.** Symbols for intersection points.

**Symbols**	**Intersection Points**

*B_1_, B_2_, B_3_*	three circles
*P_1_, P_2_*	two intersection points of *B_1_* and *B_2_*
*Q_1_, Q_2_*	two intersection points of *B_2_* and *B_3_*
*R_1_, R_2_*	two intersection points of *B_1_* and *B_3_*
*P, Q, R*	three vertices of the triangle
